# Improvement of loperamide-hydrochloride-induced intestinal motility disturbance by *Platycodon grandiflorum* polysaccharides through effects on gut microbes and colonic serotonin

**DOI:** 10.3389/fcimb.2023.1105272

**Published:** 2023-03-13

**Authors:** Mengqi Hao, Jing Song, Xiaohu Zhai, Nuo Cheng, Cong Xu, Shuangying Gui, Juan Chen

**Affiliations:** ^1^ College of pharmacy, Anhui University of Chinese Medicine, Hefei, Anhui, China; ^2^ MOE-Anhui Joint Collaborative Innovation Center for Quality Improvement of Anhui Genuine Chinese Medicinal Materials, Hefei, Anhui, China; ^3^ Anhui Province Key Laboratory of Pharmaceutical Preparation Technology and Application, Hefei, Anhui, China

**Keywords:** platycodon grandiflorum polysaccharide, intestinal motility disturbance, 5-hydroxytryptamine, gut microbiota, constipation

## Abstract

Constipation is a common gastrointestinal symptom characterized by intestinal motility disorder. The effects of *Platycodon grandiflorum* polysaccharides (PGP) on intestinal motility have not been confirmed. We established a rat model of constipation induced by loperamide hydrochloride to elucidate the therapeutic effect of PGP on intestinal motility disorder and to explore the possible mechanism. After PGP treatment (400 and 800 mg/kg) for 21 d, PGP clearly relieved gastrointestinal motility, including fecal water content, gastric emptying rate, and intestinal transit rate. Moreover, the secretion of motility-related hormones, gastrin and motilin, were increased. Enzyme-linked immunosorbent assay, western blot, immunohistochemistry, and immunofluorescence results confirmed that PGP significantly increased the secretion of 5-hydroxytryptamine (5-HT) and the expression of related proteins, such as tryptophan hydroxylase 1, 5-HT4 receptor, and transient receptor potential ankyrin 1. 16S rRNA gene sequencing showed that PGP significantly increased the relative abundance of *Roseburia*, *Butyricimonas*, and *Ruminiclostridium*, which were positively correlated with 5-HT levels. However, the relative abundance of *Clostridia_UCG-014*, *Lactobacillus*, and *Enterococcus* were decreased. PGP improved intestinal transport by regulating the levels of 5-HT, which interacts with the gut microbiota and the intestinal neuro-endocrine system, further affecting constipation. Overall, PGP is a potential supplement for the treatment of constipation.

## 1 Introduction

Chronic constipation is a heterogeneous gastrointestinal (GI) condition that predominantly includes functional constipation and irritable bowel syndrome with constipation (IBS-C) (Camilleri et al., 2017). Patients with constipation exhibit symptoms of difficult or infrequent defecation or a sense of incomplete defecation, which may be accompanied by abdominal pain and bloating. The persistence and refractory nature of constipation bring great psychological pressure to patients and threaten their quality of life. Moreover, it is also deemed to be a risk factor for colon cancer (Kyle et al., 2021). A global systematic review and meta-analysis revealed that estimates of the prevalence of chronic constipation ranged from 2.4% to 35% globally (Suares and Ford, 2011). Moreover, the incidence of chronic constipation tends to increase with aging, which imposes a substantial public health burden and has future economic consequences (Pinto Sanchez and Bercik, 2011).

The exact etiology and pathophysiology of chronic constipation are incompletely described. However, multifactorial etiology involving diet, genetic susceptibility, drug side effects, impaired colon motility, and an alteration in neurotransmitters in the gut has been described(Forootan et al., 2018). Previous studies have suggested the complex microbial communities are harbored in the gut and functional GI disorders have reciprocal effects (Zhang et al., 2021). Recent clinical studies have reported that alterations in the gut microbial profile are linked to the symptoms of constipation (Ge et al., 2017). An analysis of the intestinal microbiota of patients with constipation showed a decrease in the number of beneficial bacteria and incremental species diversity (Tian et al., 2021). Given its role in microbe-host crosstalk, the gut microbiota has been related to the pathogenesis of chronic constipation. The effects of the gut microbiota on host biology include modulating systemic metabolism, immunity development, altering GI motility, shaping intestinal physiology, and brain–gut communication (Lynch and Pedersen, 2016). Although intestinal dysbiosis is a key plausible risk mechanism for chronic constipation, there is conflicting evidence about how the composition of the gut microbiota varies in patients with constipation. Advances in precise mechanism-based microbiota therapies for chronic constipation may be the right direction.

The intestinal epithelium serves as a critical interface in host-microbe interactions. Enteroendocrine cells (EECs) with sensory functions are distributed along the entire digestive tract. They may be evoked by a diverse range of enteric stimuli, including microbes or microbial metabolites derived from digested nutritive materials. Stimulated EECs transmit microbial stimuli by secreting hormones or neuronal transmitters, which exert local or systemic effects on host physiology *via* downstream pathways. Several studies have shown that stimulating EECs with microbial metabolites improves intestinal peristalsis through 5-hydroxytryptamine (5-HT) secretion(Bunnett, 2014; Reigstad et al., 2015; Yano et al., 2015). Thus, therapeutic regimens based on the intestinal flora and gut neurotransmitters have become a potential strategy for treating constipation.

Currently, constipation is most commonly managed with laxatives, secretagogues, and colonic prokinetics. However, the side-effects of drug treatments for constipation are difficult to control, especially those for laxatives, which lead to drug dependence and electrolyte disorders(Roerig et al., 2010). Natural polysaccharides are classically known for their ability to improve the intestinal microecology by modulating microbes and microbial metabolites. For instance, polysaccharides from *Chrysanthemum morifolium* and *Spirulina* platensis increase the abundance of beneficial bacteria, and decrease the abundance of harmful bacteria, resulting in improvements in functional constipation (Ma et al., 2019; Wang et al., 2021). Moreover, some natural polysaccharides, such as *Enteromorpha intestinalis* polysaccharides(Li et al., 2022) and *Nostoc sphaeroides* Kütz polysaccharide(Liu et al., 2021b), have been reported to have the function of up-regulating 5-HT, thus regulating intestinal peristalsis. Polysaccharides from enteromorpha were proved to increase 5-HT4 receptor expression in distal colon as so to enhanced intestinal motility(Ren et al., 2017). *Platycodon grandiflorum* is a daily dietary material and traditional medicine commonly used as a dietary ingredient in Asian countries, such as China and North Korea. Notably, the main active ingredient of *P. grandiflorum* is polysaccharides. Previous researches on PGP have mainly focused on antioxidant effects and immunomodulating activity (Sheng et al., 2017; Pang et al., 2019). A recent study showed that PGP regulate the imbalance in the intestinal microbiota caused by particulate matter 2.5 exposure (Shan et al., 2022). However, there is no data showing that PGP ameliorates intestinal motility disturbances by regulating the gut microbiota.

In this study, PGP was extracted and characterized by Fourier transform-infrared spectroscopy (FT-IR) and ion chromatography (IC). We investigated the therapeutic effects of PGP on loperamide-hydrochloride-induced constipation in rats. 16S rRNA gene sequence analysis and pathology methods were used to assess improvement of loperamide-hydrochloride-induced intestinal motility disturbance by *Platycodon grandiflorum* polysaccharides through effects on gut microbes and colonic serotonin.

## 2 Materials and methods

### 2.1 Materials

*P. grandiflorum* (Jacq.) A. DC. was purchased from Hefei Mandi New Co., Ltd (Hefei, China), and were identified and stored at the College of Pharmacy, Anhui University of Chinese Medicine. Loperamide hydrochloride was purchased from Xi’an Janssen Pharmaceutical Ltd (Xi’an, China). Activated charcoal was purchased from Shanghai RichJoint Chemical Regents Co., Ltd. (Shanghai, China). The remaining chemicals and solvents were all of analytical grade.

### 2.2 Chemical identification of PGP

PGP was isolated by hot water extraction and 85% ethanol/water precipitation, according to a previously described method (Shan et al., 2021). Using the phenol-sulfuric acid method, we determined that the total sugar content of PGP was 89%. Next, to characterize the PGP, FT-IR spectra, with a frequency ranging from 4,000 to 400 cm^−1^ were collected using an FT-IR spectrometer (Thermo Electron, Waltham, MA, USA). Finally, IC was used to determine the monosaccharide composition of the polysaccharides. Briefly, after hydrolysis, the sample solution (10 mg) and standards were diluted to 10 ppm and added to ampoules. The sample solution and mixed standard were analyzed using IC with an electrochemical detector, using a Dionex Carbopac TMPA20 column (3*150) with 15 mM NaOHC: 15 mM NaOH & 100 mM NaOAC, eluted at a flow rate of 0.3 mL/min at 30°C.

### 2.3 Animal experimental design

**Figure 1:**
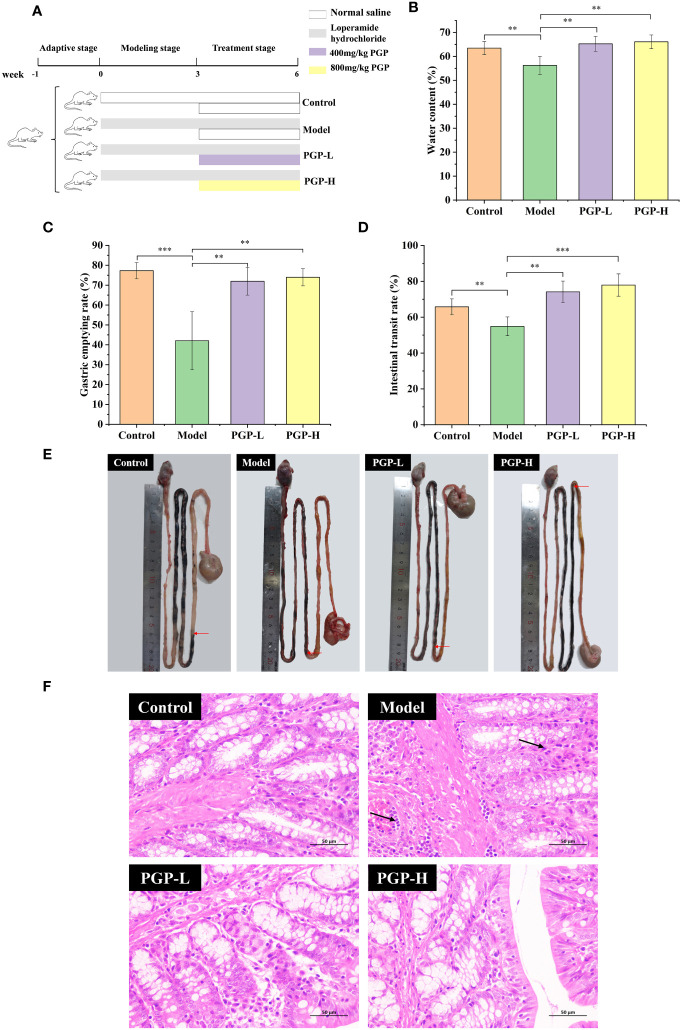
Effect of PGP on excretion parameters and pathological detection of colon in rats. **(A)** Animal experiment flow chart. Effect of PGP on gastrointestinal motility, based on **(B)** fecal water content, **(C)** gastric emptying rate, and **(D)** intestinal transit rate. **(E)** Visual observation was performed to assess intestinal transit. **(F)** The hematoxylin and eosin staining method was used to analyze the colon sections of rats under light microscope. Red arrows mean the location of the charcoal propulsion in small intestinal, and black arrows mean inflammatory cell infiltration. **p< 0.01, ***p< 0.001.

Male specific-pathogen-free Sprague Dawley rats (200 ± 20 g) were purchased from Huaxing Experimental Animal Farm (Zhengzhou, China) with the license key, SCXK(YU)2019-0002. All animal experiments were approved by the Anhui University of Chinese Medicine Animal Experiment Ethics Committee. The details of the animal experimental design are described in **Figure 1A**.

After 1 week of accommodation, rats were randomly assigned to the control, model, PGP at low dose (PGP-L; 400mg/kg), or PGP at high dose (PGP-H; 800mg/kg) groups (n = 5 per group). To construct a constipation model, the rats were administered loperamide by oral gavage at a dose of 3 mg/kg every day for 6 weeks (Wang et al., 2021). Simultaneously, a comparable volume of normal saline solution was administered to the control group. From the fourth week, the PGP-L and PGP-H groups were supplemented with PGP at 400 mg/kg and 800 mg/kg, respectively. Six weeks later, the rats were anesthetized with sodium pentobarbital. Subsequently, blood was drawn from the abdominal aorta, and serum was prepared by centrifugation. The colon and caecum contents of five rats from each group were placed in sterile 2 mL cryovials, frozen immediately in liquid nitrogen, and stored at -80°C for analysis of the intestinal microbiota.

### 2.4 Measurement of fecal water content

Each rat was placed in a metabolic cage after PGP treatment. Individual rat’s excrement was collected and weighed 16 h later. The feces were dried and weighed when the recording was finished. Then, using the following formula, the rat’s fecal water content was calculated.


fecal water content (%) = (dry weight of the feces/wet weight of the feces) × 100%

### 2.5 Measurement of gastric emptying rate and small intestine transport rate

Rats were fasted for 12 h before receiving an oral dose of 2 mL of a 5% activated carbon suspension to evaluate GI peristalsis. The rats were euthanized 30 min after gavage, and tissues from the stomach to the cecum were collected. The whole stomach was then weighed and emptied of its contents and the net weight of the stomach was recorded. The gastric emptying rate was calculated using the following formula.


gastric emptying rate (%) = (net stomach weight/gross stomach weight ) × 100%

Meanwhile, the entire length of the small intestine and activated carbon propulsion were measured. The distance from the pylorus to the front of the activated carbon was recorded as the distance that the activated carbon advanced in the intestine.


intestinal transit rate (%) = (carbon propulsion length/whole length of small intestine) × 100%

### 2.6 Pathological observations of the colon

Colon tissue was embedded in paraffin after being treated in 4% paraformaldehyde. It was then sectioned, stained with hematoxylin and eosin, and histopathologically analyzed. Under an upright optical microscope (Nikon, Tokyo, Japan), the changes in tissues between the four groups were examined.

### 2.7 Determination of the levels of motilin, gastrin, and 5-HT

The levels of motilin (MLT), gastrin (GAS), and 5-HT in rat serum samples were measured using commercial enzyme-linked immunosorbent assay (ELISA) kits (Shanghai Enzyme-linked Biotechnology Co., Ltd, Shanghai, China). Colon tissue was washed and then homogenized with phosphate-buffered saline (PBS; 0.2 g of tissue per 1.8 mL of PBS). The supernatant was collected for the 5-HT determination.

### 2.8 Analysis of transient receptor potential ankyrin 1 expression by immunohistochemical staining

Colon tissue was promptly fixed in 4% paraformaldehyde, embedded in paraffin blocks, and cut into thin sections (4 microns) for immunohistochemistry (IHC). The sections were dewaxed and hydrated and antigen retrieval was performed by heating. To block the activity of endogenous peroxidases, we placed the sections in 3% hydrogen peroxide for 25 min in the dark. Next, the sections were blocked with 3% bovine serum albumin (BSA) at 37°C for 30 min. Anti-transient receptor potential ankyrin 1 (TRPA1) antibody (Wuhan Servicebio Technology Co., Ltd, Wuhan, China) was added dropwise and the sections were kept overnight at 4°C. Following secondary antibody application, the tissues were incubated for 50 min at room temperature. Finally, the chromogenic solution, 3,3’-diaminobenzidine, was added and the sections were stained with hematoxylin. Positive granules were quantified using Image Pro-Plus 6.0 (Media Cybernetics, Inc., Rockville, MD, USA) and appeared as brownish yellow granules microscopically.

### 2.9 Detection of TRPA1 expression by immunofluorescence

After deparaffinization and rehydration, slides were submerged in ethylenediaminetetraacetic acid antigen retrieval buffer (pH 8.0), incubated at a sub-boiling temperature for 8 min, left to stand for 8 min, and then incubated again at a sub-boiling temperature for 7 min. The sections were then blocked with 3% BSA for 30 min, and the anti-TRPA1 antibody was added and the sections were incubated overnight at 4°C. After washing with PBS, a fluorescent secondary antibody was added dropwise and the sections were incubated at room temperature for 50 min, and then stained with 4′,6-diamidino-2-phenylindole. A spontaneous fluorescence-quenching agent was added and the sections were incubated for 5 min. Fluorescence microscopy was used to evaluate the expression of TRPA1 (green fluorescent).

### 2.10 Detection of protein expression levels of tryptophan hydroxylase 1, 5-HT4 receptor, and TRPA1 by western blotting

Radioimmunoprecipitation assay buffer (Thermo Fisher Scientific, Waltham, MA, USA) was used to lyse the colon tissue. To determine total protein expression, a bicinchoninic acid protein assay kit was used. The protein was extracted by centrifuging the protein extract supernatant at 12,000 rpm for 10 min at 4°C. Sodium dodecyl-sulfate polyacrylamide gel electrophoresis was used to separate 25 g of denatured protein, which was then transferred to polyvinylidene fluoride membranes at 25 V for 30 min. After blocking the membranes with 5% nonfat milk powder, the membranes were incubated with primary antibody overnight at 4°C.

The following antibodies were used: anti-tryptophan hydroxylase 1 (TPH1) (Zen-Bio, Durham, NC, USA; 381130, 1:1,000); anti-5-HT4 receptor (Zen-Bio, 860013, 1:1,000); anti-TRPA1 (GeneTex, Irvine, CA, USA; GTX54765, 1:1,000). Subsequently, the membranes were rinsed with Tris-buffered saline with Tween 20 and incubated for 30 min with a horseradish-peroxidase-conjugated goat anti-rabbit secondary antibody (SAB, Greenbelt, MD, USA; L3012; 1:5,000) for 30 min. Finally, an enhanced chemiluminescence system was using to detect the protein bands (Affinity Biosciences, Cincinnati, OH, USA). The band intensities were determined using Image Pro Plus software (version 6.0; Media Cybernetics, Rockville, MD, USA). All values were normalized to β-actin (Cell Signaling Technologies, Danvers, MA, USA; R23613, 1:2,000).

### 2.11 Gut microbiota analysis

Total genomic DNA from rats’ cecal contents was extracted using MagPure Soil DNA LQ Kit (Magen, Guangzhou, China) according to the manufacturer’s instructions. The quality and quantity of DNA was verified using a NanoDrop spectrophotometer and agarose gel electrophoresis. An upstream primer with the sequence, 5’-TACGGRAGGCAGCAG-3, and a downstream primer with the sequence, 5’-AGGGTATCTAATCCT-3, were used to amplify the V3 and V4 regions of the 16S rRNA gene. Polymerase chain reaction was performed using specific primers, barcodes, and Ex Taq high-fidelity enzyme (Takara, Kusatsu, Japan). The amplicons were then purified and pooled for paired-end sequencing on a MiSeq platform (Illumina, San Diego, CA, USA). 16S rRNA gene amplicon sequencing was performed by OE Biotech Co., Ltd. (Shanghai, China). Raw sequencing data were in FASTQ format. First, Cutadapt software was used to cut out the primer sequence for the raw data sequence. Then DADA2 was used to perform quality control analysis of the qualified double-ended raw data in the previous step according to the default parameters of QIIME 2 (2020.11), such as filtered low quality sequences, denoised, merged and detect and cut off the chimera reads, to obtain representative sequences. QIIME2 software package was used to select the representative sequences of each ASV, and all the representative sequences were compared with the database. Silva (version138) database was used for 16S rRNA gene comparison.

### 2.12 Statistical analysis

Data are presented as the mean ± standard error of the mean for each group. Comparisons between two groups were performed by Student’s t-test using SPSS Version 26.0 (SPSS Software, Chicago, IL, USA). *p*< 0.05 was considered as statistically significant. The outcomes for alpha diversity and the relative abundance of gut microbiota at phylum level and genus level are expressed as median ± interquartile range (IQR).

## 3 Results

### 3.1 Characteristics of PGP

**Figure 2:**
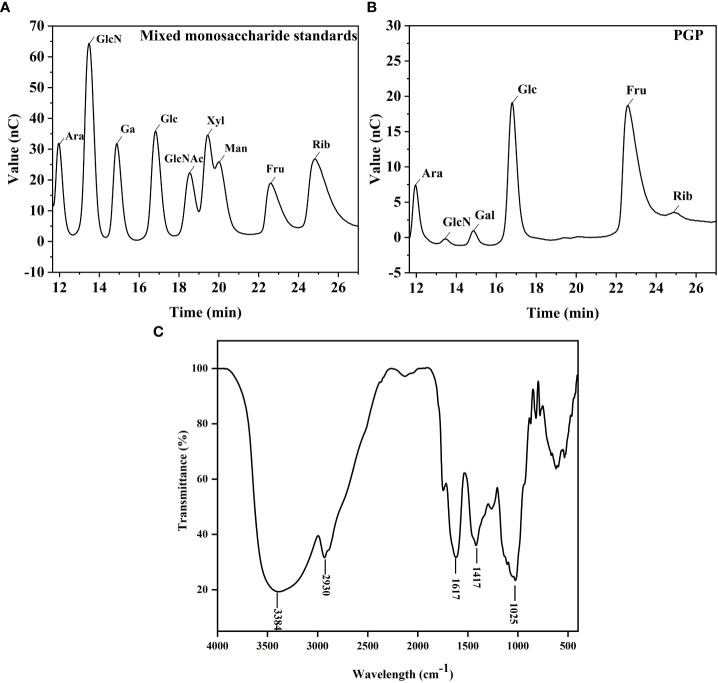
Structural characterization of PGP. Ion chromatography chromatograms of **(A)** standard monosaccharides and **(B)** PGP; **(C)** infrared chromatogram of PGP.

As shown in **Figures 2A, B**, the monosaccharide composition of PGP was determined using IC. PGP was found to be mainly composed of fructose and glucose, with a molar ratio of 80.6% and 11.9%, respectively. The FT-IR spectrum of PGP displayed the characteristic peaks of polysaccharides (**Figure 2C**). The strong, broad absorption band at 3,384 cm^-1^ was attributed to the O-H stretching vibration (Wang et al., 2017). The weak absorption peak at 2,930 cm^-1^ was the characteristic peak of C-H and was caused by the stretching vibration of a methylene group (Lemieszek et al., 2022). The strong absorption peak at 1,617 cm^-1^ suggested the telescopic vibration of C=O (Yang et al., 2015). The medium-strong absorption peak at 1,417 cm^-1^ was ascribed to the variable angle vibration of C-H (Zheng et al., 2014).

### 3.2 Effect of PGP on excretion parameters

To investigate the therapeutic effect of PGP on functional constipation, rats were administered PGP for 3 weeks. As expected, the rats treated with loperamide had markedly decreased fecal water content, intestinal propulsion rate (*p*< 0.01, vs. control group), and gastric emptying rate (*p*< 0.001, vs. control group), suggesting that PGP treatment contributed to dry stools and suppressed GI motility. Thus, the model of constipation was successfully created. After the administration of PGP-L and PGP-H, the abovementioned indicators were reversed (*p*< 0.01). In particular, PGP-H significantly increased the intestinal transit rate in rats with constipation (*p*< 0.001; **Figures 1B–E**). These results indicated that PGP had a particular curative effect on functional constipation.

### 3.3 Effect of PGP on colon pathology

Subsequently, to demonstrate the influence of PGP on pathology, we examined the pathological effects of PGP on colonic tissues. In the control group, the colonic tissue structure of the rats was complete and clear, and the goblet cells were arranged neatly, with no abnormal lesions. However, in the model group, the infiltration and aggregation of inflammatory cells were observed in the submucosa of colonic tissues, and the cell boundaries were vague. After PGP treatment, inflammatory infiltration of the tissue was reduced, with the cells arranged neatly (**Figure 1F**). Of note, PGP-H showed slightly greater effects than PGP-L. These results indicated that PGP is critical for protecting the intestinal structure.

### 3.4 Impacts of PGP on the levels of GI hormones

**Figure 3:**
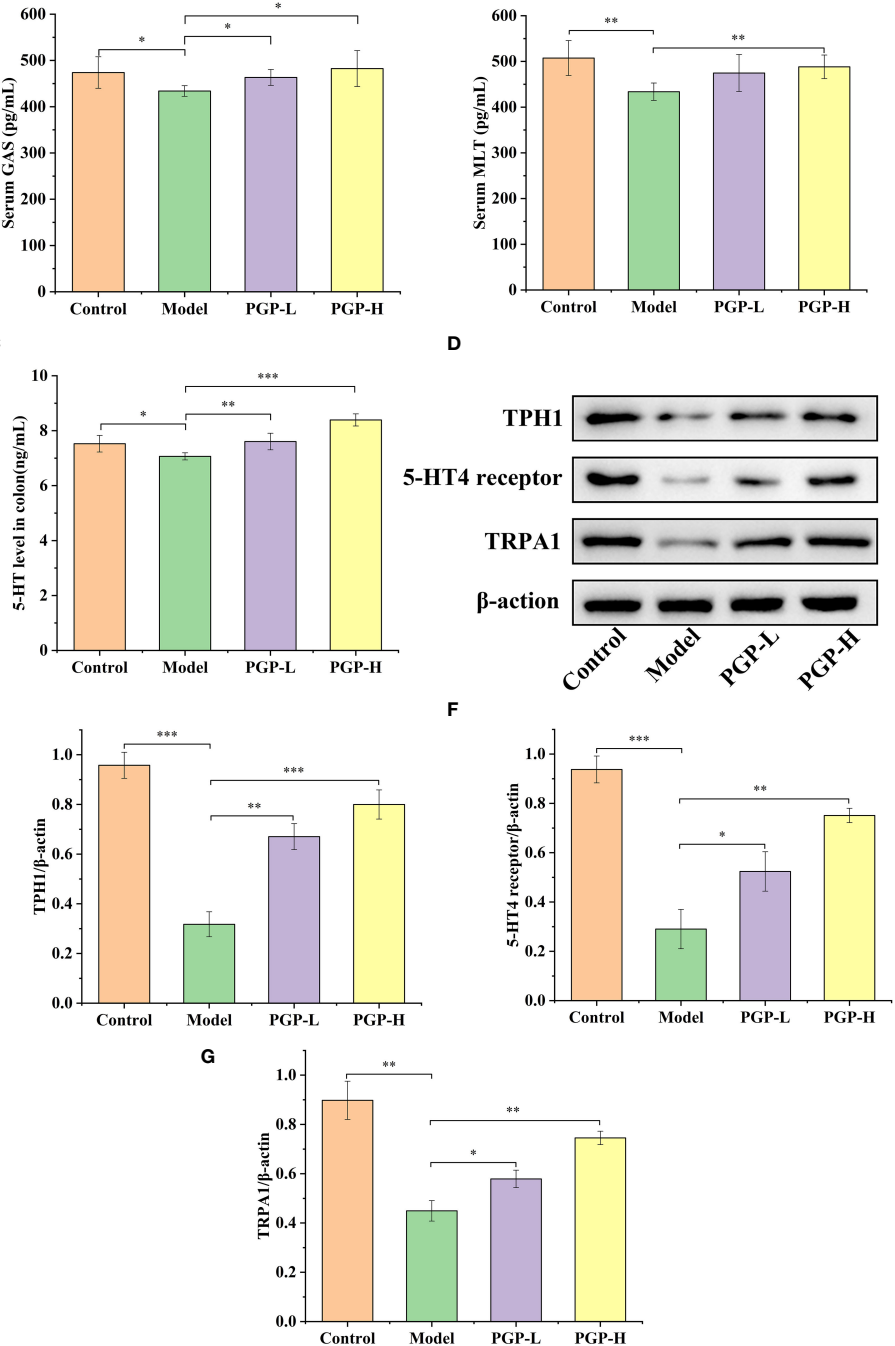
Effect of PGP on the levels of gastrointestinal hormones. **(A)** Gastrin (GAS) levels in serum; **(B)** motilin (MLT) levels in serum; **(C)** 5-hydroxytryptamine (5-HT) levels in the colon. **(D)** Expression levels of tryptophan hydroxylase 1 (TPH1), 5-HT4 reporter, and transient receptor potential ankyrin 1 (TRPA1) were detected by western blotting. Relative abundance of **(E)** TPH1, **(F)** 5-HT4 receptor, and **(G)** TRPA1. *p< 0.05, **p< 0.01, ***p< 0.001.

Furthermore, as a crucial element in modulating digestive function, we detected the levels of GI hormone release in the blood and colon. As shown in **Figures 3A–C**, rats in the model group showed lower levels of MLT, GAS, and 5-HT (*p*< 0.05, vs. control group), indicating that loperamide-induced constipation affected the secretion of related hormones. On the contrary, the GAS levels in serum were higher after PGP treatment (*p*< 0.05, vs. model group). In addition, PGP-H treatment significantly in-creased the levels of the MLT (*p*< 0.05, vs. model group). There are close links between 5 -HT and constipation. In this study, we found that the levels of 5-HT were distinctly higher after PGP-L (*p*< 0.01) and PGP-H (*p*< 0.001) treatment than in the model group.

### 3.5 Expression of TPH1, 5-HT4 receptor, and TRPA1

**Figure 4:**
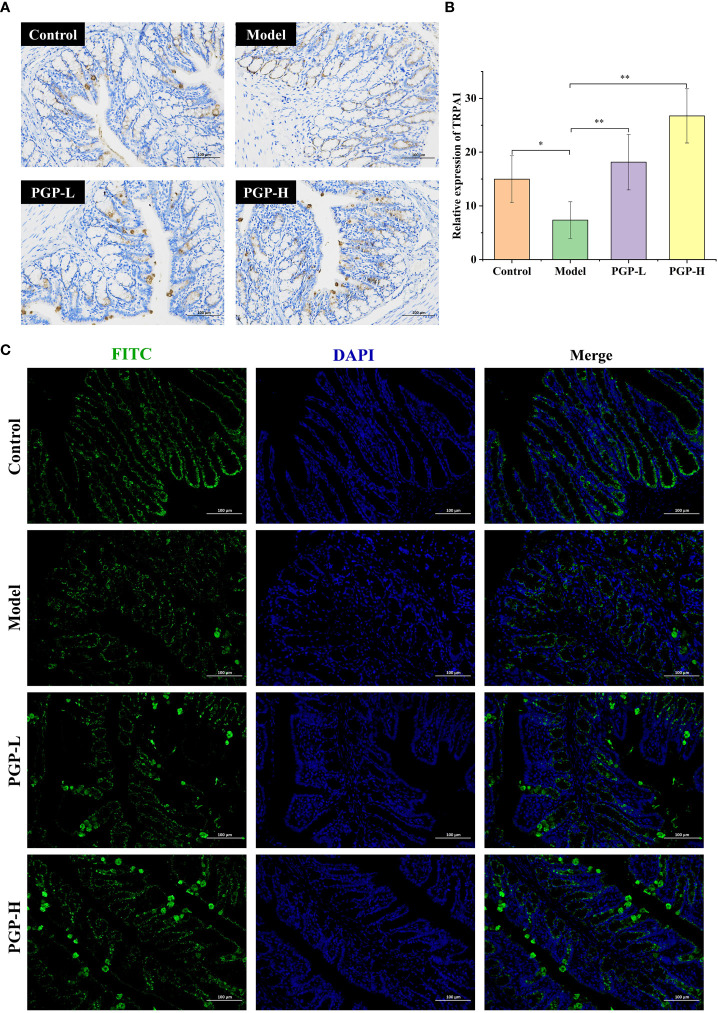
Expression of TRPA1 in colon tissue was detected by **(A)** immunohistochemistry and **(C) **immunofluorescence; **(B) **relative abundance of TRPA1. *p< 0.05, **p< 0.01. FITC, fluorescein isothiocyanate; DAPI, 4′,6-diamidino-2-phenylindole.

We previously tentatively showed that ingesting PGP increased the levels of 5-HT. To further verify the effect of PGP on the generation and consumption of 5-HT, we investigated the rate-limiting enzyme (TPH1), and 5-HT4 receptor at the protein level by western blotting (**Figure 3D**). Loperamide-hydrochloride-induced rats showed significant increases in TPH1 and 5-HT4 receptor levels compared to rats in the control group (*p*< 0.001). In contrast, PGP-L (*p*< 0.01) and PGP-H (*p*< 0.001) activated TPH1 expression in a dose-dependent manner (**Figure 3E**). We also observed a downtrend of 5-HT4 receptor in the Model group compared the PGP-L (*p*< 0.05) and PGP-H (*p*< 0.01) group (**Figure 3F**). Additionally, TRPA1 activation has the capacity to increase 5-HT synthesis. We used IHC, immunofluorescence, and western blot to determine the expression level of TRPA1 in the colon. Compared with the model group, the expression of TRPA1 was augmented by PGP-L and PGP-H treatment in **Figures 3G**, **4A–C** (*p<*0.05 and *p<*0.01). Therefore, we hypothesized that TRPA1 was a mediator of the regulatory effects of PGP on 5-HT release.

### 3.6 Regulation of the intestinal microbiota by PGP

**Figure 5:**
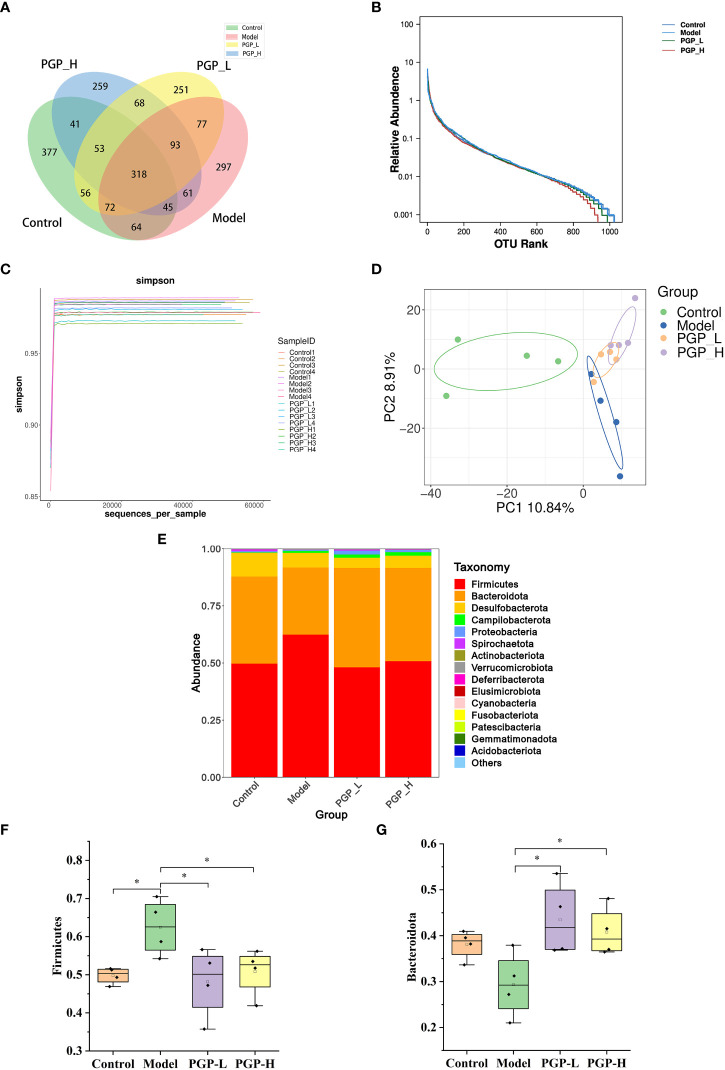
Regulation of the intestinal microbiota by PGP on in rats with constipation. **(A)** Venn diagram of the number of common and operational taxonomic units (OTUs) in the flora in the cecum of rats; **(B)** rank-abundance curve; **(C)** amplicon sequence variant/diversity index dilution curve; **(D)** principal co-ordinate analysis of gut microbiomes; **(E)** cumulative histogram of species at the phylum level. Relative abundance of **(F)** Firmicutes and **(G) **Bacteroidetes. *p< 0.05.

The gut microbiota is considered to have one of the most important roles in regulating intestinal conditions. Through sequence optimization, a total of 1,030,719 reads were generated for 16 samples from the four groups, and were then clustered. A total of 2,329 operational taxonomic units (OTUs) were shared among the four treatment groups, while the control, model, PGP-L, and PGP-H groups had 377, 297, 251, and 259 unique OTUs, respectively (**Figure 5A**). In addition, the rank-abundance curve was flat, indicating high sample uniformity (**Figure 5B**). The amplicon sequence variant/diversity index dilution curve flattened out with an increase in the number of extracted sequences, demonstrating that the sequencing data volume was reasonable to reflect biological information (**Figure 5C**). The α-diversity, based on Chao1 and Shannon indices, was used to reflect species richness and diversity, respectively. At the OTU level, there was no obvious difference in α-diversity among all the groups (**Figures S1A, B**). β-diversity was used to assess ecological similarity and distance. Therefore, we performed a distance-based main co-ordinate analysis at the OTU level. The variations in microbial composition are shown in **Figure 5D**. The control group is on the left of the figure, whereas the model group moved to the center when constipation was induced, indicating that loperamide altered the composition of the gut microbes. The PGP-L and PGP-H groups are on the right, and were significantly different from the model group. In particular, the structure of the fecal microbiota in the PGP-H group was clearly changed.

To further investigate which bacterial communities were distinct among groups, at the phylum level, we analyzed the similarity of bacterial taxa. The results of community structure analysis showed that Firmicutes and Bacteroidota were the main flora, with Firmicutes accounting for the largest proportion (**Figure 5E**). The abundance of Firmicutes was markedly increased in the model group (*p*< 0.05) and decreased in the PGP-L and PGP-H groups, compared with the control group (*p*< 0.05). However, the abundance of Bacteroidetes notably increased in the PGP-L and PGP-H groups (*p<* 0.05), but decreased in the model group (**Figures 5F, G**).

**Figure 6:**
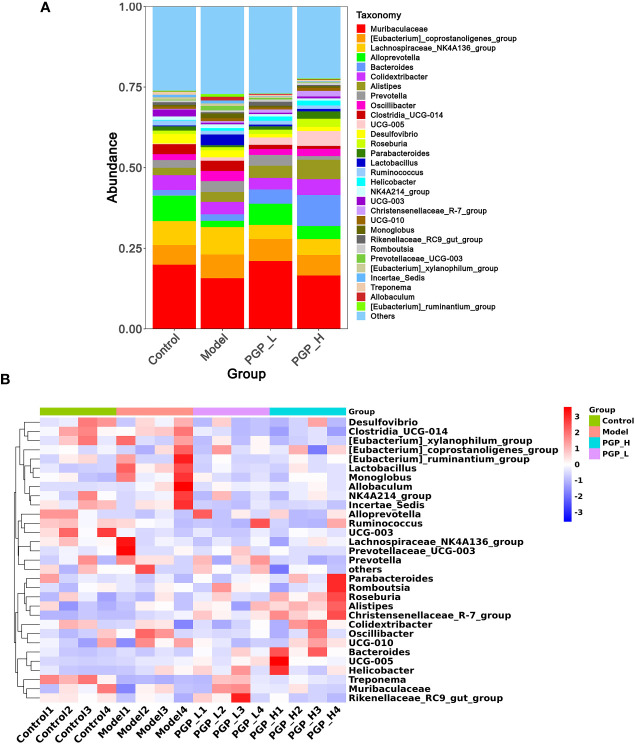
Structure of the gut microbiota at the genus level. **(A)** Cumulative histogram of species at the genus level. **(B)** Clustering heat map of species at the genus level.

**Figure 7:**
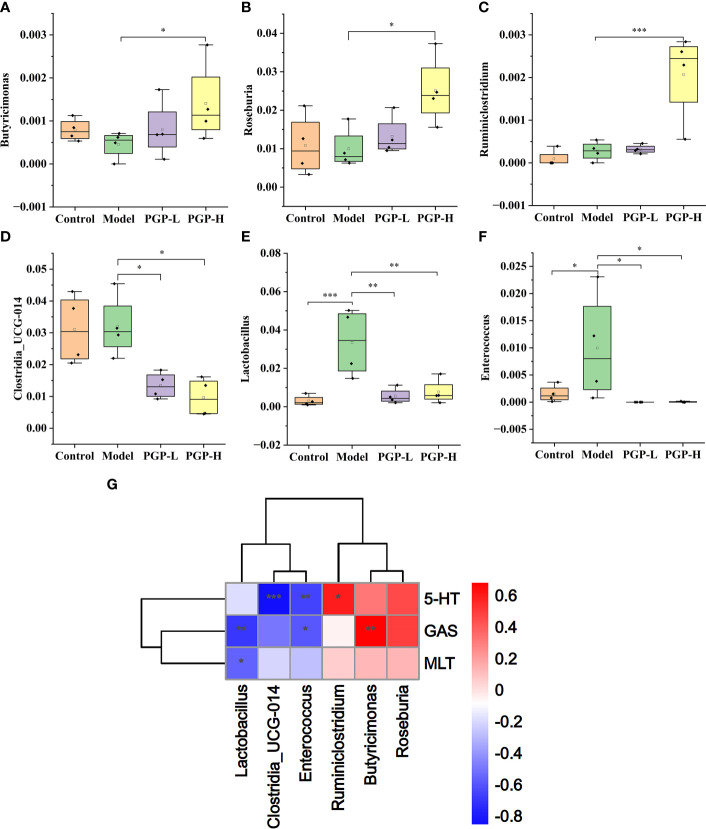
Relative abundance of **(A)** *Butyricimonas*; **(B)** *Roseburia*; **(C)** *Ruminiclostium*; **(D)** *Clostridia UCG-014*; **(E)** *Lactobacillus*; and **(F)** *Enterococcus*. **(G)** Spearman’s correlation analysis of the intestinal microbiota and constipation-related physiochemical parameters in a heatmap. *p< 0.05, **p< 0.01, ***p< 0.001.

**Figure 8:**
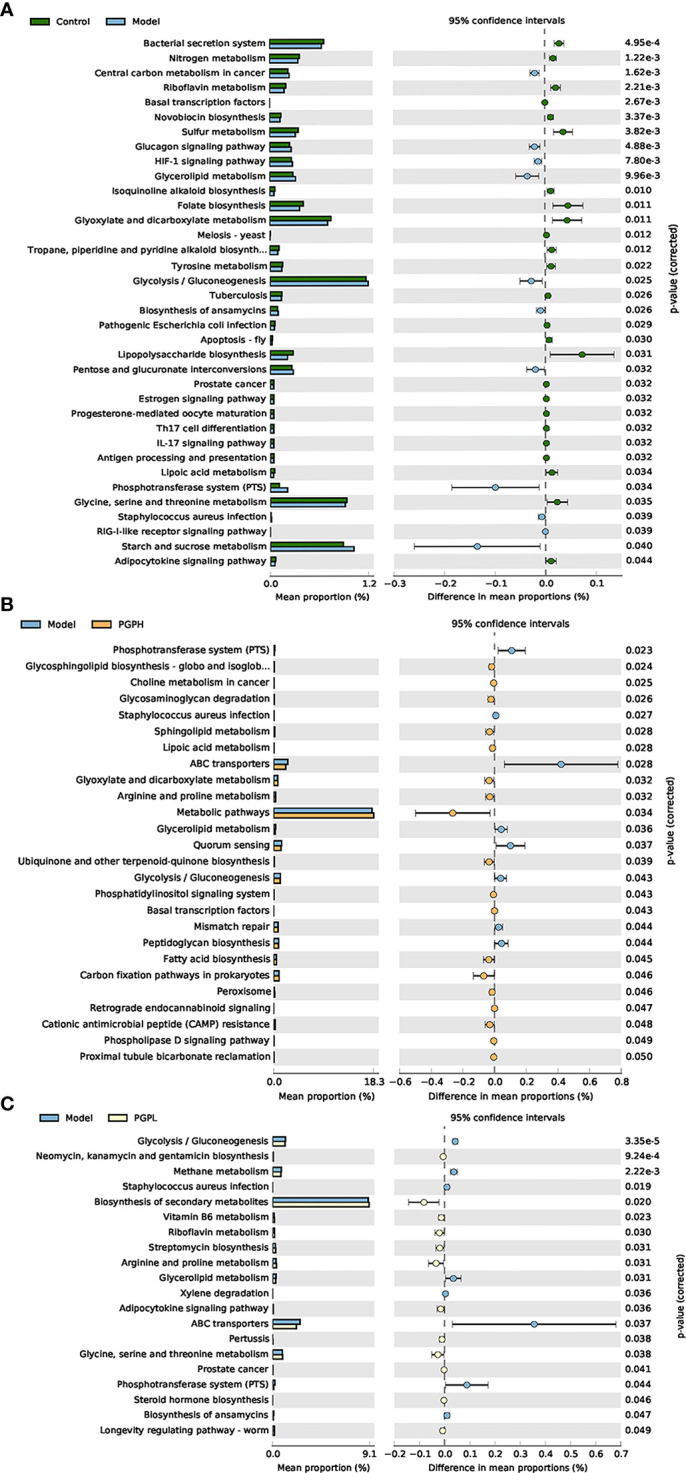
Functional prediction of altered gut microbiota, based on Kyoto Encyclopedia of Genes and Genomes pathways using the phylogenetic investigation of communities by reconstruction of unobserved states analysis. **(A)** Control group versus model group; **(B)** model group versus PGP-H group; **(C)** model group versus PGP-L group.

Next, we investigated the bacterial composition at the genus level to further evaluate the structure of gut microbiota. A cumulative histogram and heat map analyses were employed to track genus-level variations in the gut microbiota among the four groups (**Figures 6A, B**). The results demonstrated an increasing trend in the abundance of *Roseburia* (*p*< 0.05), *Butyricimonas* (*p*< 0.05), and *Ruminiclostridium* (*p*< 0.001), in PGP-H group compare with their abundance in the model group, with no discernible difference between the model and control groups (**Figures 7A–C**). In addition, the administration of ropivacaine hydrochloride decreased the abundance of *Lactobacillus* (*p*< 0.001) and *Enterococcus* (P< 0.05). Conversely, the PGP-L and PGP-H groups showed a significant downward trend in *Clostridia_UCG-014* (*p*< 0.05), *Lactobacillus* (*p*< 0.01), and *Enterococcus* (*p*< 0.05) when compared to the model group (**Figures 7D–F**). A correlation heatmap was then plotted using Spearman’s correlation analysis to determine how the gut microbiota of rats and characteristics linked to constipation were related (**Figure 7G**). The abundance of *Lactobacillus*, *Clostridia_UCG-014*, and *Enterococcus* was negatively correlated with 5-HT, GAS, and MLT levels, while the abundance of *Roseburia*, *Butyricimonas*, and *Ruminiclostridium* was positively correlated with 5-HT, GAS, and MLT levels. In particular, the correlations between 5-HT levels and *Clostridia_UCG-014* (*p*< 0.001), *Enterococcus* (*p*< 0.01), and *Ruminiclostridium* (*p<* 0.05) abundance were statistically significant. To determine whether the changes in cecal content flora in different experimental groups affected their metabolic activities, a phylogenetic investigation of communities by reconstruction of unobserved states (PICRUSt) analysis was conducted. In the model group, starch and sucrose metabolism, the phosphotransferase system, glycerolipid metabolism, and glycolysis/gluconeogenesis were strongly upregulated in response to loperamide treatment, whereas lipopolysaccharide biosynthesis, folate biosynthesis, and glyoxylate and dicarboxylate metabolism pathways were down-regulated (**Figure 8A**). An up-regulation of the biosynthesis of secondary metabolites was observed in the PGP-L group, and metabolic pathways were up-regulated in the PGP-H group (**Figures 8B, C**). These results revealed that the consumption of loperamide hydrochloride damaged the structure of the gut microbiota, whereas the administration of PGP appeared to alleviate these effects. In summary, PGP played a crucial function in shaping the composition of the gut microbiota.

## 4 Discussion

Constipation is one of the most prevalent GI conditions globally, and it has negative effects on the lives and psychological health of patients. Guided by security, effectiveness, and expenditure, dietary interventions, particularly dietary fiber intake, may be the first-line treatment. As a dietary fiber, PGP has been shown to have gut therapeutic properties and prebiotic activity. We hypothesized that PGP is a candidate to maintain the intestinal equilibrium. In the clinic, loperamide is a conventional anti-diarrheal drug, which induces constipation *via* activation of opioid receptors, thereby prolonging the intestinal transit time (Mackerer et al., 1976). In this regard, loperamide has commonly been used in animal models, and a loperamide-induced constipation model in rats was established in this study. There is a large amount of water in the feces waiting for GI absorption when intestinal peristalsis is slow. At the same time, the low rate of gastric emptying also laterally reflects the inhibition of GI peristalsis. Here, the gastric emptying rate, transit time, and water content of the feces gave direct evidence for the laxative effects of PGP. Our study revealed that the abovementioned symptoms were all significantly altered following PGP administration, ultimately leading to the stimulation of defecation.

EECs make up 1% of all intestinal epithelial cells, representing the largest endocrine organ (Latorre et al., 2016). GI hormones are a group of peptides secreted by endocrine cells, and they make essential contributions to regulating GI function. The G cells of the gastric antrum produce the majority of the GAS, which acts on parietal cells to promote acid secretion and preserve the gastric epithelium’s structural and functional integrity (Walsh, 1990; Sanger and Lee, 2008). MLT stimulates the motility of the GI tract. The release of MLT assists in the removal of undigested matter from the GI tract and guards against bacterial over-growth in the upper gut (Sanger, 2008). In our study, PGP accelerated the secretion of GAS and MLT, and partially restored GI function.

5-HT is a prominent neurotransmitter that is highly abundant in the gut (ac-counting for approximately 95% of the total 5-HT (Gershon, 2013)), and is a driving force of the peristaltic reflex (Heredia et al., 2013). 5-HT is synthesizing by EECs scattered throughout the whole GI tract, and is released in response to chemical and mechanical stimuli. 5-HT mediates GI functions, such as peristalsis, secretion, and pain perception, by binding to 5-HT-specific receptors (Liu et al., 2021a). Besides, IBS-C patients have lower 5-HT levels in colonic mucosal cells (Coates et al., 2004), which is consistent with the findings of our study. In addition, PGP reversed the decreasing trend in 5-HT levels. TPH1 is a rate-limiting enzyme that plays a regulatory role in the synthesis of 5-HT, such that the amount of TPH1 directly determines the amount of 5-HT. In addition to this synthesis step, the binding of 5-HT to its receptor is also an essential part of its role. Neurons in the central and peripheral nervous systems, particularly those in the enteric nervous system, express 5-HT4 receptors, which are G protein-coupled receptors. In isolated guinea pig colon tissue, desensitization of 5-HT4 receptors interferes with propulsive motility, supporting the hypothesis that the 5-HT4 receptor and peristalsis are related (Grider, 2006). As such, prucalopride, a 5-HT4 receptor agonist, has been recognized as a therapeutic option for constipation. We found that the 5-HT4 receptor was markedly activated after PGP treatment. The abovementioned findings indicate that PGP specifically ameliorated constipation by accelerating the whole process from the synthesis to the utilization of 5-HT. Based on these findings, 5-HT is a unique signaling molecule that plays an irreplaceable role in the regulation of intestinal peristalsis. Thus, 5-HT is a possible target for the treatment of intestinal peristalsis disorders. Further research is required to confirm this.

To further explore the mechanism for the 5-HT-mediated treatment of constipation, we studied TRPA1. TRPA1 is a non-selective cation channel of the TRP super-family that serves as a sensor molecule, mediating the release of 5-HT and thereby, regulating GI motility (Nozawa et al., 2009). Strikingly, TRPA1 activator administration has been found to effectively decrease the intestinal transit time in mice(Kojima et al., 2014). Furthermore, paeoniflorin has the ability to alleviate loperamide-induced constipation by mediating G-protein-coupled bile acid receptor/TRPA1 signaling. In our study, the expression of TRPA1 was activated by PGP (Zhan et al., 2021).

The gut microbiota is closely related to physiological processes, including immune maturation, neurological signaling, intestinal endocrine processes, and metabolism (Lynch and Pedersen, 2016). Germ-free (GF) mice have been shown to display shorter GI transit time after reconstituting the gut microbes (Yang et al., 2017). The gut microbiota has been shown to be instrumental in the regulation of 5-HT secretion. The levels of 5-HT and TPH1 in the colon are markedly lower in GF mice than specific pathogen-free mice. Even so, colonic 5-HT levels have been shown to return to normal following treatment, confirming that the absence of 5-HT in GF mice is microbiota-dependent (Yano et al., 2015). Additional research has indicated that gut microbiota metabolites trigger the production of 5-HT through the TRPA1 channel, and that 5-HT, in turn, stimulates intestinal motility (Ye et al., 2021).

In our experiments, although PGP administration did not significantly change the abundance or α-diversity of the gut microbiota in rats, the β-diversity results indicated that PGP shifted the composition of the gut microbiota in a dose-dependent manner. Compared with the model group, the PGP group had lower abundance of Firmicutes and a higher abundance of Bacteroidetes phyla. The Firmicutes phylum has been shown to be predominant in fecal samples from IBS-C subjects (Zeber-Lubecka et al., 2016), which is consistent with our findings. Furthermore, at the genus level, PGP enriched the relative abundance of butyrate-producing bacteria, including *Roseburia*, *Butyricimonas*, and *Ruminiclostridium*, while suppressing several pernicious bacteria, such as *Clostridia UCG-014*, *Enterococcus*, and *Lactobacillus*. Butyrate is produced by the decomposition indigestible carbohydrates and provides energy for colonocytes (Louis and Flint, 2009). More importantly, there is evidence that the stimulation of butyrate increases intestinal motility by affecting both the rhythm of the colonic motor complex and the peristaltic reflex (Tan et al., 2020). In addition, butyrate promotes intestinal 5-HT production by enhancing the transcription of TPH1 (Reigstad et al., 2015). Therefore, we speculated that there is an association between the increase in colonic 5-HT content and the enrichment of these bacteria in the intestinal tract. *Clostridia* is a broad genus of gram-positive bacteria. It has been proposed that some *Clostridia* species possess the ability to infect hosts by releasing potent toxins, increasing the risk of enterotoxemia, necrotic enteritis, and pseudomembranous colitis (Carter et al., 2014; Zaragoza et al., 2019). *Lactobacillus* strains induce the production of early proinflammatory cytokines, such as interleukin-8, tumor necrosis factor-α, and interleukin-6, facilitating inflammatory responses by macrophages (Rocha-Ramírez et al., 2017). Moreover, *Enterococcus* infections have been associated with intestinal inflammation (Vedantam and Viswanathan, 2011). The pathology results showed obvious inflammatory infiltration in rats of the model group, and thus, we speculated that the colonization of these harmful bacteria stimulated inflammation and destroyed the intestinal mucosal barrier.

Correlation analysis identified a linked between these altered intestinal microbiota and constipation-related biomarkers. According to PICRUSt analysis, PGP affected the metabolism of the gut microbiota, demonstrating a regulatory role in gut microbial activity. Particularly, the uptake of PGP restored the phosphotransferase system induced by loperamide hydrochloride. PGP is mainly composed of fructose and glucose. Fructose and glucose are respectively transported into cells by phosphoenolpyruvate-sugar phosphotransferase (PTS) system to produce fructose-6-phosphate and glucose-6-phosphate, respectively, for glycolysis(Goh and Klaenhammer, 2015). The glycolytic pathway of colon is closely related to maintaining the integrity of intestinal wall cells and energy supply(Kovács et al., 2021). In summary, the phosphotransferase system plays a significant role in affecting the energy source of intestinal peristalsis. Above all, our study demonstrated the remodeling effect of PGP on the structure of the gut microbiota, and therefore, the peristaltic function of the intestine was promoted.

## 5 Conclusion

As mentioned above, this study is the first attempt to evaluate the effect of PGP on loperamide-hydrochloride-induced intestinal motility disorder in rats and to explore the related mechanism *in vivo* through animal experiments. The results showed that PGP significantly improved GI motility and GI hormone levels in rats. Moreover, it promoted intestinal peristalsis by increasing the expression levels of 5-HT-related proteins and mediating the composition of the intestinal flora, thereby improving constipation. Even so, the current study only discusses the correlation between intestinal flora and 5-HT, and whether the intestinal flora can play a role in promoting intestinal peristalsis by participating in the regulation of the level of 5-HT, this study has not carried out in-depth exploration and lacks verification experiments. We will establish a pseudo-sterile rat model to compare its 5-HT and intestinal motility with those of normal rats. Our findings provide new evidence for the treatment of intestinal motility disturbance with PGP, and as a potential constipation-regulating supplement, PGP has an application prospect.

## Data availability statement

The data presented in the study are deposited in the NCBI SRA, repository, accession number PRJNA908641.

## Ethics statement

The animal study was reviewed and approved by Anhui University of Chinese Medicine Animal Experiment Ethics Committee.

## Author contributions

Methodology, MH. writing – original draft, MH. data curation, MH. methodology, JS. investigation, JS, XZ, and JC. formal analysis, NC, XZ, CX. resources, JC. conceptualization, JC. writing – review & editing, JC. funding acquisition, JC, SG. project administration, JC. All authors contributed to the article and approved the submitted version.
