# Supplementary material for: Improvement of loperamide-hydrochloride-induced intestinal motility disturbance by Platycodon grandiflorum polysaccharides through effects on gut microbes and colonic serotonin
Source: Front Cell Infect Microbiol. 2023 Mar 13;13:1105272. doi: 10.3389/fcimb.2023.1105272 (PMC10040651; doi:10.3389/fcimb.2023.1105272)
Supplement: Supplementary file 1 [file DataSheet_1.docx]

Supplementary Material

## Supplementary Figures


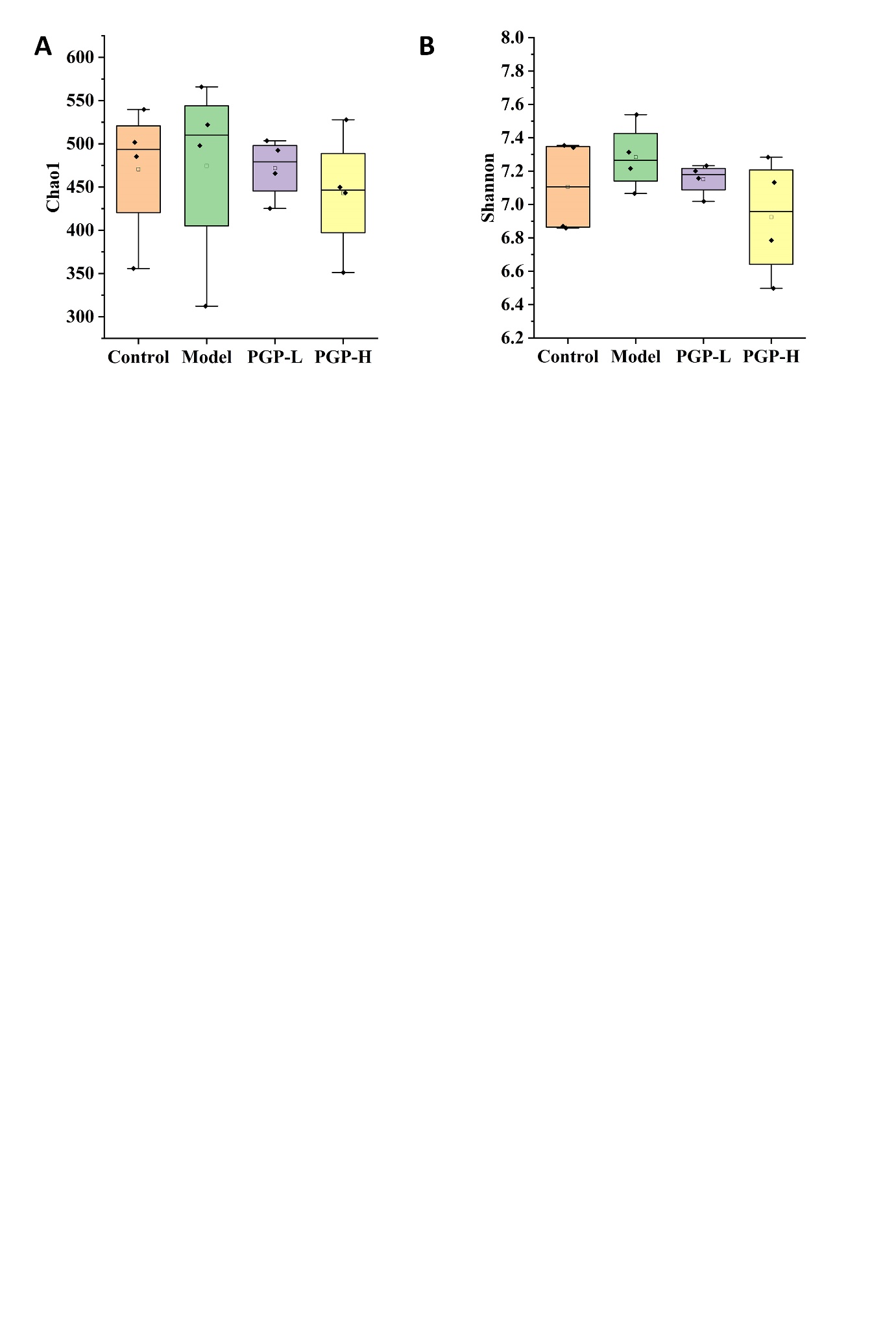


**Supplementary Figure S1**: α-Diversity based on Chao1 (A) and Shannon (B) indices.
